# Spontaneous Closure of a Fully Developed Macular Hole in a Severely Myopic Eye

**DOI:** 10.1155/2014/182892

**Published:** 2014-03-05

**Authors:** C. Bruè, I. Rossiello, J. M. Guidotti, C. Mariotti

**Affiliations:** Ophthalmology, Department of Neuroscience, Polytechnic University of Marche, Ancona, Italy

## Abstract

*Purpose.* Myopic macular holes can be difficult to close with surgery and are frequently associated with retinal detachment. We report on a case of a macular hole in a severely myopic eye that underwent spontaneous closure. *Methods.* An observational case study. *Results.* A 55-year-old female was referred to Ophthalmology for a central scotoma and metamorphopsia in the right eye. Visual acuity was 1/20 in both eyes. Fundus examination showed loss of the foveal depression, with a small yellow ring in the center of the fovea in the right eye, and a tilted optic disc and peripapillary staphyloma bilaterally. Spectral domain optical coherence tomography (SD-OCT) revealed a fully developed macular hole with a rim of thickened and slightly elevated retina in the right eye. The patient refused surgery. After 4 years of follow-up, her visual acuity improved to 20/40 in the right eye, and SD-OCT revealed spontaneous sealing of the macular hole without bare retinal pigment epithelium. *Conclusions.* Myopic macular holes represent a challenge regarding their management, and the prognosis is often poor.

## 1. Introduction

Macular holes are rare in the general population, although they are found as a common complication in severely myopic eyes, and can be associated with retinal detachment [1]. Foveal detachment often precedes the formation of a macular hole in severely myopic eyes [2]. A small percentage (6.3%) of severely myopic eyes develop a macular hole in the absence of visual symptoms [1]. Enlargement of the lesion, or posterior retinal detachment, can cause insurgence of symptoms such as metamorphopsia or reduced central visual acuity. The pathogenesis of macular holes in eyes that are severely myopic has been widely examined, although it is still not fully understood. Several factors have been proposed to be causative, including axial length elongation of the myopic eye, posterior staphyloma, chorioretinal atrophy, and vitreous modifications, such as posterior vitreous detachment and posterior vitreous schisis that induces anteroposterior or tangential vitreous traction [3].

Controversy also remains regarding the management and prognosis of such lesions. Macular holes vary from those that are idiopathic to those that are severely myopic. The myopic form appears to be more difficult to close with surgery and to be associated with a higher incidence of retinal detachment. Spontaneous closure of full-thickness idiopathic macular holes has been described in 3.5% of patients, at around 60 days after initial presentation and before scheduled surgery [4]. To the best of our knowledge, we provide here the first description of spontaneous closure of a macular hole in degenerative myopia.

## 2. Case

A 55-year-old Caucasian female came to our attention in a routine follow-up in 1997. Her best corrected visual acuity was 20/40 in the right eye, with a spherical equivalent of −18, and 1/200 in the left eye, with a spherical equivalent of −17. The measurements of the axial lengths using an optical biometer (IOLMaster, Carl Zeiss Meditec) gave 33.1 mm in the right eye and 32.8 mm in the left eye.

**Figure 1:**
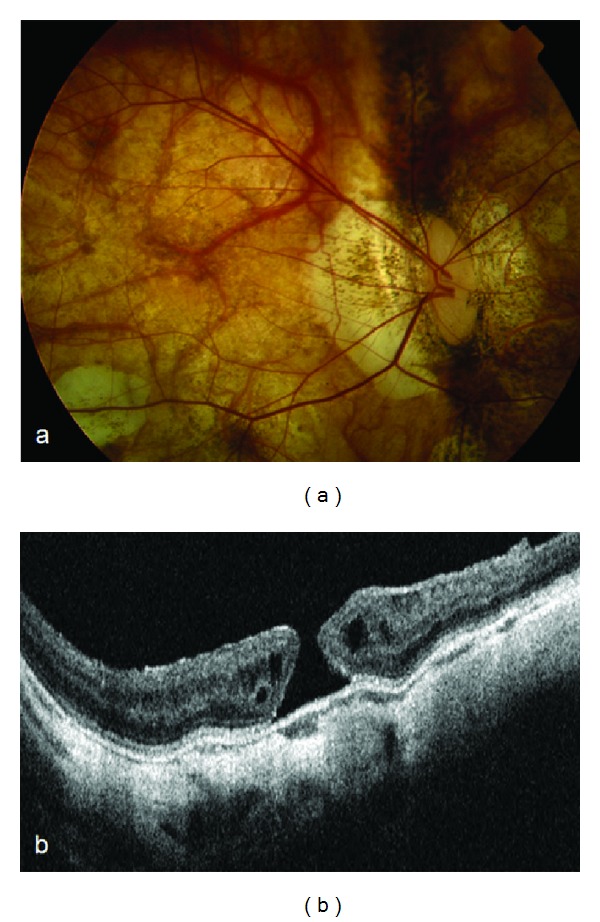
Ophthalmoscopy of the right eye shows a well-defined round foveal area that was slightly elevated with respect to the perifoveal retina (a). SD-OCT establishes a full-thickness macular hole with elevated edges of the lesion, a small accumulation of intraretinal fluid in the perilesional area, and no subretinal fluid cuff (b).

In January 2001, her visual acuity in the right eye worsened to 20/100, following corticonuclear opacity of the lens. She had phacoemulsification surgery a few months later, with good visual recovery to 20/25. Fundus examination revealed a bilateral peripapillary staphyloma and a normal macula appearance. Spectral domain optical coherence tomography (SD-OCT; Heidelberg Engineering Inc., Dossenheim, Germany) confirmed a normal foveal profile bilaterally. Two years later, the patient came back, complaining of a progressive reduction in visual acuity and metamorphopsia in the right eye. She did not report any trauma. Her best corrected visual acuity was 1/20 in the right eye. Indirect ophthalmoscopy revealed a well-defined round foveal area that was slightly elevated with respect to the perifoveal retina (Figure 1(a)). SD-OCT confirmed a full-thickness macular hole with a relatively big diameter (603 µm) and elevated edges of the lesion (Figure 1(b)). There was a small accumulation of intraretinal fluid in the perilesional area and no subretinal fluid cuff. On ultrasonography, posterior vitreous detachment was evident. The patient refused surgery.

At one month of follow-up, the best corrected visual acuity and the morphology of the macular hole were stable. At the one-year follow-up, the best corrected visual acuity was unchanged, while SD-OCT showed a reduction in the intraretinal fluid and of the diameter of the macular hole, as well as a flattening of the edge of the hole.

**Figure 2:**
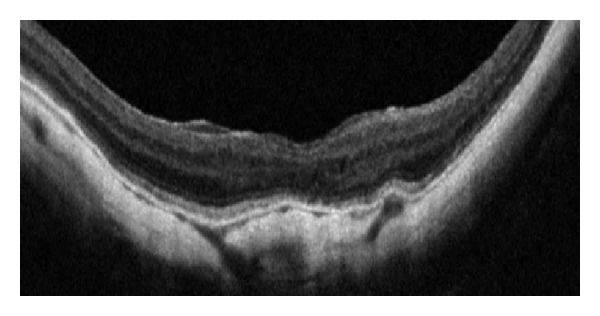
SD-OCT exhibits a flattened and reattached hole rim around the whole circumference of the previous macular hole.

Four years after the initial detection of the macular hole, the patient had a marked improvement in visual acuity, to 20/40, and absence of metamorphopsia. SD-OCT revealed a flattened and reattached hole rim around the whole circumference of the previous macular hole (Figure 2).

## 3. Discussion

Spontaneous closure of stage IV idiopathic full thickness macular holes is less prevalent and less well understood than the closure of traumatic macular holes [5]. However, to the best of our knowledge, spontaneous closure of a stage IV myopic hole has never been reported.

Several processes for spontaneous closure of idiopathic macular holes have been defined, including a bridging effect of retinal tissue and glial cell proliferation across the hole; complete detachment of the posterior hyaloids, which results in a reduction in the anteroposterior tractional forces; and formation of a contractile epiretinal membrane that causes shrinkage of the hole and cell proliferation at its base [6]. Cases of spontaneous closure of a full-thickness macular hole in one eye have been reported after vitreoretinal surgery for full-thickness macular hole in the other eye [7]. Further studies have hypothesized that the prone position and the gravitational forces implicated can promote complete detachment of the posterior vitreous, releasing the forces that contribute to hole progression [5]. Mitamura et al. [8] defined the common features for spontaneous closure of a traumatic hole as young age, small macular hole size, and posterior vitreous detachment.

In pathological myopic eyes, such as those of our patient, the sclera, choroid, Bruch's membrane, retinal pigment epithelium, neurosensory retina, and the vitreous are all affected. Bulb elongation and posterior staphyloma are the hallmark of the disorder. All of the tissues involved in the staphyloma show specific alterations, as well as retinal schisis, localized retinal detachment, and the macular hole. Examining the structural changes that occur during pathological myopia might help to understand the mechanisms and cells involved in the plugging of a myopic macular hole.

Clinicopathological reports on idiopathic and myopic macular holes that have been closed by vitrectomy have suggested that Muller cells and astrocytes are the most likely candidates for the repair of such macular holes. Okubo et al. [9] described early changes detected by SD-OCT during the spontaneous closure of an idiopathic full-thickness macular hole. They postulated that the retinal tissue protruding from the interior wall of the macular hole is at the level of the external limiting membrane (ELM), which is made of Muller cells that can proliferate and extend centripetally. Muller cells are giant cells that fill the entire thickness of the retina from the internal limiting membrane to the ELM, where they create the *zonulae adhaerentes* between photoreceptors and Muller cells, or between Muller cells. Retinal tissue bridging has been observed across a macular hole, which was followed by recovery of the ELM, with the consequent restoration of the inner segment/outer segment (IS/OS) junction line and foveal reattachment. A centripetally tractive action produced by the extension and/or proliferation of the Muller cells towards the center of a macular hole might lead to adhesion of other disrupted retinal layers, including the IS/OS junction. The connection by Muller cells might induce the recovery of the foveal detachment, by pressing the elevated tissue to the retinal pigment epithelium. This process would facilitate the photoreceptor activation and the IS/OS junction restoration.

These hypotheses are supported by the maturation of the photoreceptors that follows ELM formation during normal retinal development. We can hypothesize that similar mechanisms of retinal tissue recovery can also be applied in cases of myopic macular holes. Further support of this hypothesis comes from the detection of fibroblasts, myofibroblasts, and Muller cells in surgical specimens from idiopathic and myopic macular holes [10].

Our patient showed complete posterior vitreous detachment on ultrasonography. This probably contributed to the release of the anteroposterior traction, the consequent bridging effect of the retinal tissue and glial cell proliferation across the hole, and the formation of a contractile epiretinal membrane, which causes shrinkage of the hole and cell proliferation at its base.

There were two distinct features of our patient: the relatively old age and the large size of the macular hole that underwent spontaneous closure. As myopic macular holes are prone to risks, such as unfavorable visual outcome after surgery, it is advisable to have caution when considering vitreoretinal surgery for this group of patients.
